# Dental Malocclusion and Its Relation to the Podal System

**DOI:** 10.3389/fped.2021.654229

**Published:** 2021-06-22

**Authors:** María E. Cabrera-Domínguez, Antonia Domínguez-Reyes, Manuel Pabón-Carrasco, Ana J. Pérez-Belloso, Manuel Coheña-Jiménez, Antonio F. Galán-González

**Affiliations:** ^1^Department of Stomatology, Faculty of Dentistry, University of Seville, Seville, Spain; ^2^Red Cross Nursing School, University of Seville, Seville, Spain; ^3^Department of Podiatry, Faculty of Nursing, Physiotherapy and Podiatry, University of Seville, Seville, Spain

**Keywords:** children, dental, malocclusions, foot, posture

## Abstract

**Background and Objective:** This study analyzes the possibility that Stomatognathic and Postural systems are related by muscle chains. Malocclusion may influence the posture, contact between the foot and the ground, center of mass, footprint or vice-versa. This study aimed to verify whether there is a relationship between dental occlusion and podal system.

**Materials and Methods:** A cross-cutting, descriptive study was carried out on 409 children (222 boys and 187 girls) between 8 and 14 years old. Dental occlusion was assessed on the sagittal plane (Angle's classification) the contact between the foot and the ground and the center of mass were evaluated using a stabilometric platform.

**Results:** There was a statistically significant relationship between the plantigrade phase, the contact surface area and center of gravity. There was a prevalence of molar and canine Angle's class II malocclusion. In molar class II, an anterior center of gravity was predominant, in class I it was centered and in class III, it was posterior. There was significant correlation between malocclusions and the FPI (foot posture index) of the left foot and the height of the scaphoid in the right foot (*P* < 0.001).

**Conclusions:** Some authors agree with our results. There is still much uncertainty in terms of showing a relationship between both systems. In addition, there is scarce scientific evidence on the topic. Some kind of relationship between the two systems has been proven. Studies that evaluate a group of subjects in a longitudinal manner are necessary to enable the changes taking place in both systems to be defined.

## Introduction

There are various studies that attempt to show the relationship between the Stomatognathic Apparatus and the postural system through the different muscle chains (1–3). Some hypotheses suggest a relationship in both the cranial-caudal direction as well as the podo-cranial direction, therefore malocclusion and its treatments may influence body posture, contact between the foot and the ground, center of mass, footprint, and vice-versa, although it is a controversial topic among the scientific community (4–7).

It seems clear that among the structures of the Stomatognathic System and the forces it exerts on the oral cavity and in particular the teeth, there must be perfect equilibrium. Occlusion disorders may affect the rest of the Stomatognathic System and vice versa. Also, disorders in the structures and functions that maintain correct posture, faults in the posture, and/or parameters may be transmitted to other parts of the body (8, 9). Dental malocclusion is defined as the disturbances resulting from the alignment between the dental arches. Angle proposed a classification of occlusion and malocclusion based on the anteroposterior position of the first molar and the position of the canines (10, 11). According to the WHO, malocclusion is an oral disorder common in children (the second most common after cavities) and adults (the third most common after cavities and periodontal diseases).

The body is subject to the force of gravity, of which the center or center of mass is the point through which the axis of the body passes and where all the parts of which it consists (bones, tendons, muscles and joints) are balanced. When the posture is correct, the line of gravity or central axis of the body passes through the middle cervical vertebrae, in front of the dorsal vertebrae and the middle lumbar vertebrae (12). Agonist and antagonist actions, gravity and the muscular anti-gravitational function are involved in maintaining posture. This requires coordination of the muscles, maintenance of equilibrium under static and dynamic conditions, of the visual field, auditory vestibular system, and kinesthetic sensitivity (12, 13).

The transmission of false information by one of the postural control systems may negatively influence the behavior of other systems, therefore pathologies related to disorders of the cervical column, pelvis, posture or balance may affect different parts of the body in an ascending or descending manner. In the same way, any disorder in any component of the Stomatognathic System may affect occlusion and posture. Depending on the muscle chains that are activated, the cranium may take an anomalous position, overloading the cervical column and with a poor position at dorsal level to compensate. A deviation in the dental midline or displacement of the lower maxilla may determine the presence of cervical scoliosis, dorsal scoliosis in the opposite direction, and lumbar scoliosis in the same direction as the cervical scoliosis. These are disorders that may reduce vertebral arterial blood supply, cause pain in the upper limbs, reduce muscular strength or alter the gait.

In a case of Angle's class II or III, the children adopt postures that may compensate for their mandibular protrusion or retraction to obtain postural equilibrium (9, 14, 15). In class II (distocclusion), the maxilla is in the mesial position in relation to the mandibular arch and the body of the mandible is in a distal position in relation to the maxillary arch, resulting in the child bringing their head forward, affecting the TMJ, vertebral column and the center of mass. In class III (mesiocclusion), the mandible is in a mesial position in relation to the maxilla and the child positions their head to the back, affecting the vertebral column, center of mass, and general posture (16).

When maintaining balance and posture, the foot represents the first link of the kinesthetic chains. It is the functional unit that stabilizes the rest of the locomotor system during walking through contact with the ground; it is a segment that is highly adaptable and flexible, which is the first receiver and transmitter of impacts, tensions, and compressions. The surface that has contact with the ground and the area of separation of both feet form the basis for balance. Children with physiological walking often have normal occlusion, without overload injuries to the TMJ or vertebral column and are often connected with suitable posture (12, 16–19). Therefore, the body must be considered as an interconnected whole and not as a group of independent systems. In this manner, we must tackle disease in a multidisciplinary manner to avoid therapeutic failings in both directions. As a result, the aim of this study is to determine whether there are relationships between dental malocclusion and foot biomechanics.

## Methods and Study Population

### Study Design and Population

A cross-cutting descriptive study was carried out, the sample for which was 409 children (222 boys and 187 girls) between 8 and 14 years old, selected during the 2018–19 academic year (20). The study was performed in various schools in the city of Seville. The randomized sample included education centers, using as the selection criteria the geographical proximity to the Faculty of Dentistry. In order not to interfere with the school timetable, we established small groups for the examination. Informed consent was requested from the parents or legal guardians. This study was approved by the Biomedical Research Ethics Committee (code number: 048412019) of the Andalusian Regional Government (20). The study was carried out according to the Helsinki Declaration on ethical principles for research involving human subjects (21). Firstly, a questionnaire (designed by the authors) was provided to the parents or guardians on the oral and postural habits of the child, the presence or absence of prior traumas, orthodontic treatments prior to the study or the use of insoles (22). The inclusion criteria were: being between 8 and 14 years old; returning the parents' survey; lack of orthodontic treatment; and lack of oral or systemic conditions that may affect the results. The exclusion criteria were: having undergone operations on the lower extremities or upper part of the body; presenting proven disorders in terms of the vertebral column; having had orthodontic treatment; having had any trauma that has modified the posture; and lacking the teeth necessary to determine the Angle's classification.

### Methods

With natural light (WHO recommendation) and usual dental equipment for an oral examination (mirrors, probes, depressors, antiseptic solution, gloves, masks, and calipers) we analyzed the molar and canine occlusion in the sagittal dimension according to Angle's classification: Angle's class I molar (normal occlusion), when the mesio-vestibular cusp of the first upper molar overlaps the vestibular sulcus of the first lower molar; class II when it does so in front and class III, when it is behind. The study data were collected by direct observation of the oral cavity, by an experienced orthodontist with more than 32 years of experience in the fiel private clinic, who was blinded to the podiatric results. The reliability of the examiner was confirmed by intraexaminer repeat examinations for 50 subjects. The intrarater ICC was 0.93 to 0.98.

In class I canine, the cusp of the upper canine overlaps the inter-dental point of contact between the canine and the first lower premolar. In class II, it does so in front of the point and in class III behind. The posture, contact between the foot and the ground and center of mass (static and dynamic) were analyzed using a stabilometric platform. We assessed the height of the scaphoids, the right and left foot posture index (FPI) and the position of the hips using a hip level. Further were evaluated posture, truncation of the scaphoids and plantar pressures. The oral data were collected by direct observation by an experienced orthodontist who did not know the podiatric results. The podiatric data, in turn, were collected by an expert podiatrist who did not have access to the oral data. The data were collected in files that were reviewed daily by a third-party reviewer.

The biomechanical examination was carried out by a principal researcher using FPI as a method that has been validated and recommended by various authors (23). This index evaluates the multi-segmental nature of the foot posture in the three planes, and does not require the use of specialist equipment. Each item on the index is scored between −2 and +2, therefore the possible total varies from −12 (very supinated) to +12 (very pronated). The measurements for this index were obtained at the beginning of the study to classify the results as supinated (−12 to −1), neutral (0 to +5), pronated (+6 to +8) or over-pronated (+9 to +12). The participants were evaluated in a relaxed position, with the foot on a bench at a height of 50 cm to facilitate the measurement. A value from 0 to +5 is considered to be a neutral position, from +6 to +12 as a pronated position and from −1 to −12 as supinated (24–26). The truncation of the scaphoids is calculated by dividing the height of the scaphoids by the truncated length of the footprint. This scaphoid measurement has had the greatest correlation with the angular measurements taken by X-ray (23).

**Figure 1:**
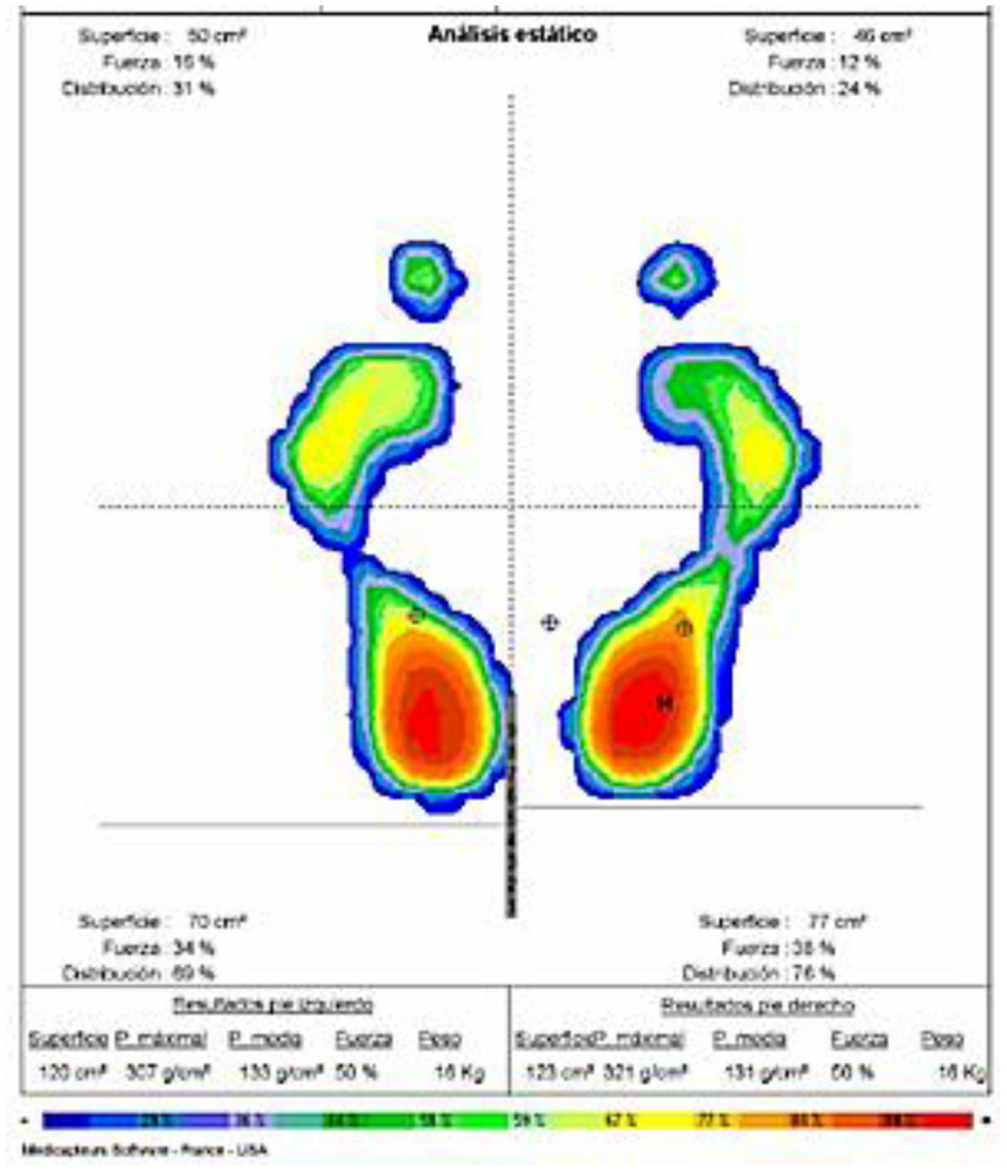
Baropodometric analysis of pressures and center of gravity.

Regarding the plantar pressures, baropodometric analysis allows us to find out the distribution of the loads or pressures in various plantar zones and thereby evaluate the direct influences of the forces applied in the three periods of the support phase, its intensity and duration (24, 25). The baropodometric measurements were carried out using the Neo-plate® pressure platform. This is a platform with a usable surface of 40 cm × 40 cm and a flat surface that is just 8 mm thick. It has resistive sensors that do not require calibration. The data were sent digitally to a computer and then processed using software that shows the parameters of plantar pressure (kPa), contact time(s), and cadence (steps/minute). The device recorded the distribution of the plantar pressure in the first and fifth metatarsal head and the calcaneus under both static and dynamic conditions. The measurement was taken twice in order to make the data more precise (Figure 1) (23).

### Statistical Analysis

The data were processed in the IBM SPSS v.26 for the statistical study. We applied the Chi-squared test on the qualitative variables and the Anova test to the following variables: FPI, truncation of the scaphoids, plantar pressures and Angle's classification in normal conditions; otherwise the Kruskal–Wallis test was applied. The quantitative variables were expressed as averages and standard deviations and the qualitative (non-numerical) variables as frequencies and percentages. The degree of relationship of the data and the strength of the association were assessed. The effect size was calculated using Cramer's V. The bivariate relationship between plantar pressures was determined using Pearson's correlation and if it was not found to be normal, Spearman's test was applied. The level of significance was established as *p* < 0.05.

## Results

The sample consists of 409 participants (222 boys and 187 girls); a strong bilateral correlation was evident between FPI, scaphoid height, overall contact force, and contact surface. Regarding the hip position, 51.2% showed a higher iliac crest on the right and only 17.6% had anteversion of the left iliac crest. Following a descending line, regarding the evaluation for genu varum/valgum, we observed that 80% had genu valgum. Finally, the participants presented the following types of feet: 37.9% had pes valgus, 45.2% had cavus valgus and 16.9% were normal. Regarding the position of the feet, data were presented in the normal range (FPI neutral).

In relation to the hip position, a relationship was found between the measurements obtained on the foot both on a barometric level (static and dynamic) as well in terms of positioning (FPI and scaphoid height). The barometric data for both feet under static and dynamic conditions and the degree of association between the FPI, scaphoid height, and pressure are shown (Table 1).

**Table 1:** Anthropometric data and foot measurements.

	**Average**	**SD**	**95% CI**	**Correlation**
Age	10.85	1.46	10.71–10.98	
BMI Kg/m^2^	18.07	3.10	17.74–18.37	
Right FPI	5.02	2.29	4.80–5.25	0.000
Left FPI	4.80	2.28	4.58–5.09	*r* = 0.884
Right scaphoid height	2.57	0.77	2.45–2.69	0.000
Left scaphoid height	2.58	0.73	2.49–2.66	*r* = 0.883
Right overall force	53.12	6.41	50.32–51.84	0.000
Left overall force	49.71	6.49	48.15–49.67	*r* = −0.997
Pressure 1st right metatarsal	136.09	124.17	121.62–150.57	0.000
Pressure 1st left metatarsal	226.45	133.12	210.93–241.98	*r* = 0.512
Pressure 5th right metatarsal	339.06	116.35	321.49–347.17	0.000
Pressure 5th left metatarsal	115.70	111.40	98.94–125.46	*r* = 0.356
Pressure right heel	471.45	155.95	453.26–489.63	0.000
Pressure left heel	530.84	144.85	511.86–545.43	*r* = 0.291
Right heel support phase	97.65	51.42	88.81–100.48	0.000
Left heel support phase	91.98	53.17	83.95–98.05	*r* = 0.427
Right plantigrade phase	264.71	102.08	252.90–276.53	0.000
Left plantigrade phase	259.21	88.02	249.01–269.40	*r* = 0.470
Right propulsion phase	341.54	83.60	331.86–351.22	0.000
Left propulsion phase	345.88	83.30	336.24–355.53	*r* = 0.524
Right contact surface	63.14	16.51	59.87–63.61	0.000
Left contact surface	58.35	17.18	55.44–59.38	*r* = 0.882

*CI, Confidence Interval; FPI, Foot Posture Index; SD, Standard deviation; r, Correlation Coefficient*.

In relation to the odontologic measurements, a predominance of Angle's class II was shown, both molar and canine, and the association was strong when compared within the same dental class and when the canine class was compared with the molar class. On the other hand, no relationship was found with the hip position and the Angle's classification (*p* > 0.05; Table 2).

**Table 2:** Oral assessment of participants.

	**Class 1**	**Class 2**	**Class 3**
Angle's classification	29.1%	60.80%	10%
molar right	*N* = 119	*N* = 249	*N* = 41
Angle's classification	21.3%	70.2%	8.6%
molar left	*N* = 87	*N* = 287	*N* = 35
Angle's classification	46.5%	43.8%	9.8%
canine right	*N* = 190	*N* = 179	*N* = 40
Angle's classification	40.3%	49.9%	9.8%
Canine left	*N* = 165	*N* = 204	*N* = 40
	**Normal**	**Hypotonic**	**leporine**
Upper lip tonicity	95.08%	3.2%	1.0%
	*N* = 392	*N* = 13	*N* = 4
Lower lip tonicity	72.1%	27.9%	0.0%
	*N* = 295	*N* = 114	*N* = 0
	**Oral**	**Nasal**	**Mixed**
Type of respiration	2.4%	67.7%	29.8%
	*N* = 10	*N* = 277	*N* = 122
	**Average**	**SD**	**95% CI**
Upper overjet (mm)	0.78	1.44	0.65–0.92
Mandibular overjet (mm)	0.15	1.09	0.002–0.28
Anterior open bite	0.02	0.15	0.002–0.04
Deviation from the midline	0.51	1.24	0.32–0.66
	***X***^**2**^	**Cramer's V**	
Right molar-Left molar	0.000	0.811	
Right molar-right canine	0.000	0.559	
Left molar-left canine	0.000	0.703	
Right canine-left canine	0.000	0.847	

*CI, Confidence Interval; SD, Standard deviation; X^2^, Pearson Chi-Square*.

### Inferential Analysis

No statistical differences were found by gender (*p* = 0.745), age (*p* = 0.097), and nationality (*p* = 0.124). An assessment was carried out under both static and dynamic conditions to deduce the possible relationships between the stomatognathic system and the foot biomechanics (Table 3). In our analysis of the variables for the foot in comparison to the Angle's classification, there was no evidence of a relationship between the FPI and the truncation of the scaphoids and the Angle's classification, in *p* ≥ 0.05. There is a relationship only between the FPI of the left foot and the scaphoid height of the right foot.

**Table 3:** Inferential analysis between Angle's classification and foot biomechanics.

	**Right**	**Left**	**Right**	**Left**
	**molar-p**	**molar-p**	**canine-p**	**canine-p**
Right FPI	0.210	0.062	0.117	0.245
Left FPI	0.160	**0.034**	0.320	0.504
Right scaphoid height	0.567	0.640	**0.045**	0.142
Left scaphoid height	0.576	0.277	0.980	0.742
Right overall force	0.781	0.868	0.107	0.074
Left overall force	0.781	0.874	0.110	0.077
Pressure 1st right metatarsal	0.052	**0.020**	0.227	0.448
Pressure 1st left metatarsal	0.935	0.540	0.851	0.410
Pressure 5th right metatarsal	0.090	**0.001**	0.715	0.214
Pressure 5th left metatarsal	0.178	0.267	0.548	0.717
Pressure right heel	**0.011**	0.554	**0.042**	**0.041**
Pressure left heel	0.267	0.095	0.100	0.670
Right heel support phase	0.262	0.331	0.335	0.528
Left heel support phase	0.644	0.822	**0.007**	0.132
Right plantigrade phase	0.321	**0.006**	**0.008**	**0.022**
Left plantigrade phase	0.259	**0.020**	**0.050**	**0.027**
Right propulsion phase	0.400	0.698	0.320	0.732
Left propulsion phase	**0.001**	**0.004**	**0.003**	0.170
Right contact surface	**0.013**	**0.041**	0.190	0.283
Left contact surface	**0.001**	**0.010**	**0.030**	**0.006**
Center of Gravity	**0.0001**	**0.0001**	**0.0001**	**0.0001**

*Kruskal Wallis Test. The statistically significant differences are shown in bold letters*.

Regarding the barometric analysis in comparison with the dental classification, a significant discovery is made with the contact surface, the plantigrade phase and especially with the center of gravity. The intensity of the association between the center of gravity and the Angle's classification was analyzed (Table 4). A predominance of anteriority in the center of gravity is shown in Angle's class II, whereas in class I the center of gravity is posterior, the same as occurs in class III. Regarding the strength of the association, we found a moderate level.

**Table 4:** Relation between center of gravity and Angle's class.

	**Center of gravity anterior *N* (%)**	**Center of gravity posterior *N* (%)**	**Center of gravity central *N* (%)**	**Association strength—^£^**
**Right molar**				
Class 1	26 (21.84)	19 (15.96)	**74 (62.18)**	*p* = 0.000
Class 2	**234 (93.9)**	11 (4.5)	4 (1.6)	0.449
Class 3	4 (9.8)	**37 (90.2)**	0	
**Left molar**				
Class 1	12 (13.8)	17 (19.5)	**58 (66.6)**	*p* = 0.000
Class 2	**259 (90.2)**	21 (7.3)	7 (2.4)	0.525
Class 3	1 (2.9)	**34 (97.1)**	0	
**Right canine**				
Class 1	27 (15.08)	29 (16.20)	**134 (74.86)**	*p* = 0.000
Class 2	**173 (96.6)**	5 (2.8)	1 (0.6)	0.359
Class 3	6 (15.0)	**34 (85.0)**	0	
**Left canine**				
Class 1	29 (17.57)	36 (21.81)	**100 (60.60)**	*p* = 0.000
Class 2	**192 (94.1)**	8 (3.9)	4 (2.0)	0.477
Class 3	1 (2.5)	**39 (97.5)**	0	

£ *, Cramer's V; the statistically significant differences are shown in bold letters*.

## Discussion

The study identified a greater prevalence of Angle's class II malocclusions in mixed dentition (10, 11). No statistically significant relationship was found between the FPI, truncation of the scaphoids and dental occlusion. Regarding the barometric analysis in relation to the dental occlusion, there were significant discoveries regarding the contact surface, the plantigrade phase and the center of gravity, with a prevalence of anteriority of the center of gravity in the children with Angle's class II; in class I, the center of gravity was centered, and in class III, there was a prevalence of posteriority of the center of mass; this association was of a moderate strength. This may be explained by the fact that in class II we have a protruded maxillar or a retracted mandible, which results in bringing the head forward, moving the center of mass forward. In class III, the opposite occurs, locating the head in a more posterior plane and moving the center of mass backwards. Regarding Angle's class I, there was a prevalence of a centered center of mass, as would be expected by the neutral position of the bite in the sagittal plane.

Novo et al. (26) described that in class II or III malocclusions, the children adopt positions to compensate for the mandibular protrusion or retraction, seeking postural equilibrium. Valentino et al. (27), in a study based on electromyography, detected correlations between the occlusal plane and the muscles of the plantar arches. In the same vein, Cuccia and Caradonna (28) evaluated the footprint of 84 subjects with temporomandibular dysfunction and a control group of 84 people without this disorder with a pressure platform; they observed differences in the plantar arch between both groups, although the results were not very conclusive.

According to Cuccia and Caradonna (28), there are studies that suggest that different mandibular positions favor postural changes, affecting the position of the foot's center of pressure and the stability of the gait; these findings are similar to our own, although obtained with different equipment. Chessa et al. (29) used a stabilometric platform to evaluate the postural changes in patients with cranio-cervical-mandibular disorders, before and after orthodontic treatment; they found evidence that the positioning of an orthodontic plate rebalanced the postural system. After treatment, 64% of the patients experienced remission of the pain symptoms. They concluded that this relationship must be studied from a multidisciplinary point of view for an overall therapeutic result.

Other studies have tried to correlate malocclusions with postural disorders, obtaining negative results. Perinetti et al. (2) did not find statistically significant relationships, including in the term “malocclusions” overbites and temporomandibular disorders. They found that the stomatognathic system may influence the cervical region, enabling the production of small imbalances in the posture in a statistically significant manner. Baldini et al. (30) found a relationship between vision and postural control, but not with occlusion.

### Limitations and Strengths

Among the possible limitations of our study we found that, although we used the force platform under dynamic and static conditions, perhaps a more detailed study using electromyography or movement analysis is necessary to be able to determine the relationships of these variables. Our work has provided significant data regarding barometric analysis and its relationship to the Angle's classification, with significant findings regarding the contact surface, the plantigrade phase, and the center of gravity. However, few studies have analyzed the correlation between the center of gravity and dental malocclusions; Baldini et al. (30) did so but without statistically significant results. Bracco et al. (31) carried out a posturometric and stabilometric analysis on 95 healthy subjects with a force platform to investigate the influence of Angle's malocclusions on the posture. All the subjects exhibited statistically significant variations of the posture with different mandibular positions; this result is in accordance with those of this study, as the variations of the mandibular position in the anteroposterior direction (Angle's classes) seem to be related to changes in the center of gravity.

Our study has certainly had limitations; one could be the lack of consideration of the effects of natural change in growing children, therefore we believe that a more long-term study would be needed to consider the effects of growth. It should not be forgotten that this type of design does not allow causality. Another was the lack of cephalometric analysis, and although this analysis provides information that is impossible to obtain by other means, the aim of this study was to observe dental malocclusion, not that of the skeleton, therefore we decided not to carry out X-rays, as the information on this malocclusion can be obtained in an observational manner without exposing the children to radiation. All this means that our findings must be taken with precaution, as this is a first cross-cutting study that may act as a starting point for others with different methodology and a greater number of variables to consider. As knowledge on this topic is currently very limited, this expanded focus would add a great deal of value to the results that we are presenting.

## Conclusions

In relation to dental malocclusions, a significant correlation was observed for the FPI points on the left foot and the scaphoid height on the right foot (*P* < 0.001). Comparing the dental classification with the center of mass, significant data were found in relation to the contact surface, especially in the plantigrade phase and in particular regarding the center of gravity. A predominance of anteriority of the center of gravity was found in subjects with Angle's class II malocclusion. In those with Angle's class I and III malocclusion, the center of gravity was in a more posterior situation. However, this relationship is moderate or relative. Possibly due to muscular compensation, no relationship whatsoever has been found with the other parameters studied, therefore greater and broader studies are required for a better understanding. We believe that in order to establish clearer relationships between the podal system and the stomatognathic system, larger studies with a greater number of variables are needed, which would possibly provide greater evidence of these relationships. In addition, it is necessary to perform assessments with multidisciplinary team where stomatologists, physiotherapists, and podiatrists are coordinated. This would allow a holistic view of the body where the systems and parts of the body interact. This studio opens the way to assess whether orthodontic treatment influences body posture.

## Data Availability Statement

The original contributions presented in the study are included in the article/supplementary material, further inquiries can be directed to the corresponding author/s.

## Ethics Statement

The studies involving human participants were reviewed and approved by Comité de Ética de la Universidad de Sevilla (CEUS). Written informed consent to participate in this study was provided by the participants' legal guardian/next of kin.

## Author Contributions

MC-D, AG-G, AD-R, AP-B, MP-C, and MC-J conceptualized the study and were responsible for the study design, data collection planning, and manuscript preparation. AG-G, AD-R, and MP-C performed data analysis, contributed to the interpretation of the results, and drafted the manuscript. All authors have read and agreed to the published version of the manuscript. All authors critically reviewed the manuscript and approved the final version of the manuscript for submission.

## Conflict of Interest

The authors declare that the research was conducted in the absence of any commercial or financial relationships that could be construed as a potential conflict of interest.

## Abbreviations

FPI: Foot posture index
ICC: Intraclass correlation coefficient
STROBE: Strengthening the reporting of observational studies in epidemiology
WHO: World Health Organization.
